# Targeting oncogenic microRNAs from the miR-371~373 and miR-302/367 clusters in malignant germ cell tumours causes growth inhibition through cell cycle disruption

**DOI:** 10.1038/s41416-023-02453-1

**Published:** 2023-10-03

**Authors:** Shivani Bailey, Marta Ferraresso, Luz Alonso-Crisostomo, Dawn Ward, Stephen Smith, James C. Nicholson, Harpreet Saini, Anton J. Enright, Cinzia G. Scarpini, Nicholas Coleman, Matthew J. Murray

**Affiliations:** 1 Department of Pathology, University of Cambridge, Cambridge, CB2 1QP UK; 2 Department of Paediatric Haematology and Oncology, Cambridge University Hospitals NHS Foundation Trust, Cambridge, CB2 0QQ UK; 3 Department of Paediatrics, University of Cambridge, Cambridge University Hospitals NHS Foundation Trust, Cambridge, CB2 0QQ UK; 4 EMBL-European Bioinformatics Institute (EMBL-EBI), Wellcome Genome Campus, Hinxton, Cambridge, CB10 1SD UK; 5 Department of Histopathology, Cambridge University Hospitals NHS Foundation Trust, Cambridge, CB2 0QQ UK

**Keywords:** Germ cell tumours, miRNAs

## Abstract

**Background:**

MiR-371~373 and miR-302/367 cluster over-expression occurs in all malignant germ cell tumours (GCTs), regardless of age (paediatric/adult), site (gonadal/extragonadal), or subtype [seminoma, yolk sac tumour (YST), embryonal carcinoma (EC)]. Six of eight microRNAs from these clusters contain the seed sequence ‘AAGUGC’, determining mRNA targeting. Here we sought to identify the significance of these observations by targeting these microRNAs functionally.

**Methods:**

We targeted miR-371~373 and/or miR-302/367 clusters in malignant GCT cell lines, using CRISPR-Cas9, gapmer primary miR-302/367 transcript inhibition, and peptide nucleic acid (PNA) or locked nucleic acid (LNA)-DNA inhibition targeting miR-302a-d-3p, and undertook relevant functional assays.

**Results:**

MiR-302/367 cluster microRNAs made the largest contribution to AAGUGC seed abundance in malignant GCT cells, regardless of subtype (seminoma/YST/EC). Following the unsuccessful use of CRISPR-Cas9, gapmer, and PNA systems, LNA-DNA-based targeting resulted in growth inhibition in seminoma and YST cells. This was associated with the de-repression of multiple mRNAs targeted by AAGUGC seed-containing microRNAs, with pathway analysis confirming predominant disruption of Rho-GTPase signalling, vesicle organisation/transport, and cell cycle regulation, findings corroborated in clinical samples. Further LNA-DNA inhibitor studies confirmed direct cell cycle effects, with an increase of cells in G0/G1-phase and a decrease in S-phase.

**Conclusion:**

Targeting of specific miR-371~373 and miR-302/367 microRNAs in malignant GCTs demonstrated their functional significance, with growth inhibition mediated through cell cycle disruption.

## Introduction

Germ cell tumours (GCTs) are a histologically diverse group of tumours that vary by clinical presentation, tumour histology, and clinical course. They present across all age groups, from the neonatal period into late adulthood, and arise in midline anatomical sites, both gonadal and extragonadal. Malignant GCTs are broadly divided into germinomas/seminomas (Sem) and non-germinomatous/-seminomatous GCTs (NGGCTs/NSGCTs)—the latter consisting of yolk sac tumour (YST), embryonal carcinoma (EC), choriocarcinoma (CHC), and mixed GCTs (containing more than one subtype). The GCT subtype teratoma (mature/immature) has benign/intermediate behaviour, best treated by surgery alone [1].

Since the introduction of platinum-based chemotherapy [2], most patients with metastatic malignant GCTs have excellent overall survival. However, within this patient group, there are cohorts with poor outcomes. First, International Germ Cell Consensus Classification poor-risk patients [3] still only have a progression/event-free survival of 54% despite improvements in supportive care [4]. Second, outcomes for certain patients who relapse are dismal, for example, those with platinum-resistant extracranial tumours [5] and intracranial NGGCT [6]. As a result, GCTs have the highest adult cause of average years-of-life-lost per person dying of cancer [7]. Furthermore, for the majority who are cured, current chemotherapy regimens cause significant long-term effects, including myelosuppression [8], nephrotoxicity [9], ototoxicity [10], irreversible pulmonary fibrosis [11], and second malignancies [9]. These effects are particularly debilitating in the predominantly young patient population which GCTs affect. Consequently, there remains a major need for an improved understanding of germ cell biology and the identification of new targets for potential therapeutic intervention. Such an approach will facilitate the development of novel agents that may improve survival in those with poor-risk disease and reduce late-effects in those with good-risk disease [12], across the diverse clinical GCT spectrum [13].

To this end, the first common biological abnormality identified across the full clinical spectrum of malignant GCTs related to dysregulated microRNA (miRNA) expression [14]. MiRNAs represent an abundant class of endogenous, short non-protein-coding RNAs, typically ~21-23 nucleotides (nt) in length, which post-transcriptionally regulate the expression of protein-coding genes, predominantly by messenger RNA (mRNA) destabilisation and degradation [15]. Through this mechanism, miRNAs critically regulate development and normal physiological processes [15, 16]. Regulation is primarily determined through the miRNA ‘seed’ region, comprising nucleotides in positions 1-8 (1-8nt), which bind to seed complementary regions (SCRs) in the 3’ untranslated region (3’UTR) of their mRNA targets [17]. The 2-7nt core seed sequence is considered most critical for targeting [17], but contributions from 1-6nt and 3-8nt seed sequences are also recognised [18]. A study of malignant GCTs identified that they are universally characterised by over-expression of eight miRNAs arising from just two miRNA ‘clusters’, namely miR-371~373 (at chromosomal locus 19q13.41) and miR-302/367 (4q25), when compared with a non-malignant cohort (comprising gonadal controls and teratomas) [14]. Importantly, six of these eight miRNAs share an identical 2-7nt seed region ‘AAGUGC’, and in addition, miR-371a-3p contains the identical 1-6nt sequence. Furthermore, certain malignant NSGCT subtypes associated with poor outcomes are characterised by over-expression of the ‘chromosome-19-microRNA-cluster’ [C19MC, or miR-515-526 [14]]—specifically EC [14], and in particular CHC [19, 20]. Of note, C19MC is co-located within 100 kilobases (kb) of the miR-371~373 cluster on chromosome 19 and a proportion of the 59 miRNAs contained within it also share the AAGUGC seed. The SCR corresponding to this specific seed region demonstrated enrichment, when compared with non-malignant controls, in the 3’UTRs of protein-coding genes downregulated in malignant GCTs, and importantly, pathway analysis identified that these downregulated genes were involved in important cellular processes, such as signal transduction and cell cycle regulation [14]. Of note, this work suggested that miR-371~373 and miR-302/367 over-expression plays a key role in malignant GCT tumorigenesis. Moreover, cisplatin-resistant GCT cell lines display further over-expression of miR-371~373 and C19MC miRNAs compared with their cisplatin-sensitive counterparts [21]. Together, the available evidence justifies further investigation of the functional/therapeutic role of these over-expressed miRNAs in malignant GCTs.

To explore such roles, different strategies exist for targeting these miRNAs at a genomic [e.g., clustered regularly interspaced palindromic repeats (CRISPR)/CRISPR-associated nuclease 9 (Cas9) systems [22, 23]], transcriptional [e.g., ‘gapmer’ inhibition targeting primary miRNAs, e.g., [24]], and mature miRNA level [e.g., peptide nucleic acid (PNA) [25] or locked nucleic acid (LNA) [26] approaches]. LNAs are modified RNA or DNA molecules with increased miRNA binding affinity [26]. For example, ‘tiny’ LNAs (8-10nt length) designed against seed-sharing miRNA families have been used in models of breast cancer [27], B-cell lymphoma [28], and medulloblastoma [29]. Furthermore, LNA/DNA ‘mixmers’ may be used experimentally, which balance affinity with specificity, reducing off-target effects seen with shorter, all-LNA inhibitors [30, 31]. Here, we sequentially targeted AAGUGC seed-containing miRNAs from the miR-371~373 and miR-302/367 clusters in malignant GCTs at the genomic, transcriptional, and mature miRNA level, including with LNA/DNA mixmers. Work is now warranted to explore the effects of targeting these oncogenic miRNAs in further pre-clinical studies, with the ultimate aim of improving outcomes for patients with malignant GCTs.

## Materials and methods

### Patient samples

The study was performed under Multicentre generic Children’s Cancer and Leukaemia Group (CCLG) Tissue Bank approval (East-Midlands/Derby REC reference 08/h0405/22+5, covering Biological Studies CCLG-2002-BS03 and CCLG-2020-BS02; formerly Trent-REC reference 02/4/071) and Cambridge Local Research Ethics Committee (reference 01/128) approval. Written informed consent was obtained from all subjects. Further analysis of published miRNA microarray expression profiling data was undertaken on 42 clinical samples, comprising 32 paediatric GCTs from 22 female and 10 male patients (12 YSTs, 11 seminomas, three ECs and six teratomas), two testicular seminomas from young adults and eight control samples, as described [14, 32]. One teratoma sample (MT-34) was excluded as it was derived from a mixed GCT and clustered with malignant GCTs, as described [32]. Messenger RNA (mRNA) array data for 45 clinical samples, comprising 37 malignant GCTs (17 paediatric, 20 adults) and eight non-malignant controls [14, 32], was used for clinical correlation of functional investigations in cell lines. These array data are publicly available at Gene Expression Omnibus, accession no. GSE18155.

### GCT cell lines

Four representative human malignant GCT cell lines were selected for in vitro studies, as previously described [32], namely 2102Ep (EC) [ExPASy Cellosaurus online cell line knowledge resource (https://web.expasy.org/cellosaurus/) Research Resource Identifier (RRID):CVCL_C522] [33], 1411H (RRID:CVCL_2268) [34] and GCT44 (RRID:CVCL_A346) [35] (both YST), and TCam-2 (Sem) (RRID:CVCL_T012) [36]. Three further authenticated cell lines were obtained from American Type Culture Collection (ATCC; Manassas, VA) for study. These were the EC cell line NCCIT (ATCC number CRL-2073: RRID:CVCL_1451), and two cell lines derived from placental CHC (in the absence of available GCT-derived CHC lines), specifically BeWo (ATCC CL-98: RRID:CVCL_0044) and JAR (ATCC HTB-144; RRID:CVCL_0360). All cells were cultured at 37 °C in 5% CO_2_ in appropriate medium containing 10% fetal calf serum and 1% penicillin/streptomycin, as described [32]. All cell lines were authenticated by short-tandem-repeat profiling [37] within the last 3 years and all experiments were performed with mycoplasma-free cells. Further analysis of published miRNA microarray expression profiling data was undertaken on six GCT cell lines [namely TCam-2 (Sem), 1411H (YST), GCT44 (YST), 2102Ep (EC), Tera-2 (EC/teratoma; RRID:CVCL_2777) and PA-1 (immature teratoma; RRID:CVCL_0479)], as described [14, 32].

### MiRNA microarray analysis and calculation of overall 2-7nt AAGUGC seed abundance

To calculate this, median normalised microarray expression values were calculated and summated for all miRNAs on the published array [14, 32] containing the 2-7nt seed region AAGUGC (*n* = 12 of 615 total miRNAs), for different malignant GCT subtypes and cell lines, and non-malignant control samples (gonadal controls and teratomas). Specifically, these miRNAs were from the miR-371~373 cluster (*n* = 2; miR-372-3p, miR-373-3p), the miR-302/367 cluster (*n* = 4; miR-302a-d-3p), and C19MC (*n* = 6; miR-519b-3p, miR-520a-e-3p).

### Quantitative reverse-transcription PCR (qRT-PCR) for miRNAs

Total RNA was isolated from clinical GCT samples and cell lines using TriReagent (Sigma-Aldrich, St Louis, Missouri, USA), following the protocol described [32]. Levels of miRNAs were then quantified in triplicate using Taqman qRT-PCR reagents and proprietary primer/probe assays (Applied Biosystems), as per the manufacturer’s instructions, with 25 nanograms (ng) of total RNA used for copy DNA synthesis and 2 µl of the resultant 15 µl product used for the final PCR step. Levels were calculated using the delta-delta-Ct method and normalised to RNU24, as described [14, 32].

### Calculation of individual miRNA cluster contribution to overall AAGUGC seed abundance in malignant GCT cell lines

Derived array or qRT-PCR expression values for representative miRNAs from the three AAGUGC seed-containing miRNA clusters (miR-371~373, miR-302/367, and C19MC) were summated and contributions from each cluster calculated. Specifically, these were miR-371a-3p, miR-372-3p, and miR-373-3p for miR-371~373 (*n* = 3), miR-302a-d-3p for miR-302/367 (*n* = 4), and miR-519b-3p and miR-520b-3p for C19MC (*n* = 2). For this work, miR-371a-3p was included as it contained 1-6nt AAGUGC, a seed position known to contribute to mRNA targeting [18]. For consistency, as only six of the eight 2-7nt AAGUGC seed-containing miRNAs from C19MC were present on the array, the two C19MC miRNAs listed above were selected for both platforms and proportionally scaled to represent the total of eight such miRNAs from C19MC.

### Genomic copy number determination for the miR-371~373 and miR-302/367 region

Primers to assess genomic copy number were designed using the website ‘Primer3’ (https://bioinfo.ut.ee/primer3/) [38] and ordered from Sigma-Aldrich (Supplementary Table S1). The genomic regions from which these miRNAs arise were 1098 base pairs (bp) and 544bp in length for the miR-371~373 (chromosome 19q13.41) and miR-302/367 (chromosome 4q25) clusters, respectively. C19MC (chromosome 19q13.41) was excluded from this analysis as it is not universally over-expressed in all malignant GCT subtypes [14], its genomic region is substantially larger at over 100,000 bp (and thus more challenging to comprehensively assess), and it contains at least 59 characterised miRNAs, of which only eight contain the 2-7nt AAGUGC seed [39]. The sequence for each cluster was obtained from the Ensembl Genome Browser (https://www.ensembl.org/index.html), Human Genome Assembly GrCh38.p10. Primer pairs were then screened using Primer-BLAST (https://www.ncbi.nlm.nih.gov/tools/primer-blast/), which uses the Basic Local Alignment Search Tool (BLAST) and a global alignment algorithm to avoid pairs that could result in non-specific amplification. Two sets of primers were designed per cluster, in the upstream and downstream regions. Quantitative PCR was performed on genomic DNA (gDNA) extracted from the cell lines, as described [40], with levels normalised to four established gDNA housekeeping genes (*B2M*, *GAPDH*, *18A*, and *18B*) and compared with human testicular gDNA (ThermoFisher Scientific Inc, USA) levels.

### Targeting the miR-371~373 and miR-302/367 clusters in malignant GCT cells

A number of approaches were taken in order to target miRNAs from the miR-371~373 and miR-302/367 clusters and demonstrate functional significance in malignant GCT cells. For CRISPR-Cas9 targeting of these two clusters at a genomic level, ‘gapmer’ inhibition of primary miRNA (pri-miR-302/367) transcripts, and PNA inhibition of mature miR-302/367 miRNAs (miR-302a-d), see Supplementary Methods, Supplementary Results, and Supplementary Tables S2–S4. The approach using LNA/DNA mixmer inhibitors is described below.

### LNA/DNA inhibitors targeting mature miR-302/367 cluster miRNAs (miR-302a-d-3p)

For this work, two LNA/DNA inhibitors were used, namely a 16nt, 69% (11nt) LNA content miR-302a-d-3p inhibitor (miR-302 super-family-inhibitor, ‘miR-302-SFI’; sequence: AAACATGGAAGCACTT) and a 10nt, 70% (7nt) LNA content miRNA inhibitor (‘short-SFI’; GGAAGCACTT), along with a 20nt, 70% (14nt) LNA content mismatch control (MMC; TTAACACGTCTATACGCCCA), (Exiqon, now Qiagen). Of note, due to its shorter length, the short-SFI was designed to target AAGUGC seed-containing miRNAs more widely, including those from the miR-371~373 cluster (predominantly miR-372-3p/miR-373-3p), and C19MC, in addition to the miR-302/367 cluster. Following optimisation to determine the greatest transfection efficiency with minimal toxicity, TCam-2 and 1411H cell lines received 37nM of inhibitor/MMC, and 2102Ep 50nM. Cells were seeded in 6-well plates to ensure ~40% confluence on the day (d) of transfection (d0). The transfection media, containing the cell-line specific inhibitor/MMC doses and the transfection reagent Viromer Blue (1 µl per 500 µl transfection solution), was replaced with standard media at 4–6 h post-transfection to minimise toxicity, and then subsequently changed every 24 h. Experiments were performed in biological triplicate. Cells were harvested for further experimental studies at set timepoints up until d7. Cell numbers were quantified using Trypan blue dye on a Countess automated-cell-counter, which gave live and dead cell counts, as described [32].

### Global messenger RNA (mRNA) microarray of cells transfected with miR-302-SFI

At d2, when direct mRNA changes following miRNA perturbation experiments are typically maximal [41], we undertook mRNA profiling in biological triplicate on miR-302-SFI-treated, MMC-treated, and untreated TCam-2, 1411H, and 2102Ep cells. RNA concentration and quality were assessed using SpectroStar (BMG Labtech, Aylesbury, UK) and Bioanalyser (Agilent Technologies, Cheadle, UK) machines. Microarray experiments were performed at Cambridge Genomic Services, Department of Pathology, University of Cambridge, using a species-specific Gene 2.1 ST Array Plate (Affymetrix, Wooburn Green, UK), according to the manufacturer’s instructions. Briefly, 100 ng total RNA was amplified along with in-line PolyA spike-in control RNA, using the WT PLUS amplification kit (Affymetrix). Successfully amplified samples were labelled using the GeneChip WT terminal labelling kit (Affymetrix) using in-line hybridisation controls. Plate arrays were processed on the GeneTitan instrument (Affymetrix) with GeneTitan ‘Hybridization, Wash, and Stain’ kit (Affymetrix). Samples were hybridised to the array, washed, stained, and scanned using the array-specific parameters provided by Affymetrix to generate raw CEL files, which underwent basic visual quality control using Command Console Viewer (Affymetrix). The resultant CEL files were loaded in the statistical language *R* using the oligo package from Bioconductor [42]. Data quality was assessed through the generation of control probe plots, boxplots, MA, and intensity distribution plots. Variation within biological replicates was investigated using clustering methods. The raw data were then pre-processed using the Robust Multichip Analysis method [43]. The data were background corrected, quantile normalised, and summarised. Following pre-processing, comparisons were performed using *limma* and results corrected for multiple-testing using the false discovery rate method [44]. Raw microarray data from these analyses is deposited at EBI Array Express (https://www.ebi.ac.uk/biostudies/arrayexpress), accession no. E-MTAB-13323.

### Assessing effects of miR-302-SFI treatment on global mRNA levels using *Sylamer*

As each miRNA can target hundreds of mRNAs, changes in expression levels of individual mRNAs following miRNA perturbation are very subtle [16]. Accordingly, such multiple shifts in mRNA expression levels are best assessed by global and pathway analyses. Thus, in brief, *Sylamer* [45] was used to assess enrichment and/or depletion of nucleotide ‘words’ of specific length (SCRs) complementary to elements of the seed region of miRNAs of interest within the 3’UTRs of genes within ranked lists, as described [45], derived from miR-302-SFI-treated, MMC-treated, and untreated cells. The output was visualised as a landscape plot of *p* values for each SCR (*y*-axis), plotted against the ranked gene list (*x*-axis), and segregated into ‘bins’ containing 200 genes in each [14, 45]. The derived single summed significance score (SSSS) was an integration of *Sylamer* significance scores for different elements that comprised the SCR and served as an overall evaluation of the enrichment or depletion of nucleotide sequences [32]. For this work, the scores were calculated by combining the *Sylamer* results for four SCR elements, all complementary to the key 2-7nt seed region of miR-302a-d-3p, namely one hexamer (2-7nt GCACTT), two heptamers (1-7nt AGCACTT and 2-8nt GCACTTA) and one octamer (1-8nt AGCACTTA). Following the generation of SSSS landscape plots, a change-point detection algorithm was employed to identify the most appropriate enrichment peak for selecting gene lists for further analyses. This algorithm computed a change-point delta value (CPDV) for each bin of 200 genes, based on the difference between its –log_10_(*p* value) and the minimum –log_10_(*p* value) across the next five bins (progressing from left to right). CPDV curves were plotted for the SCR of interest (corresponding to the 2-7nt AAGUGC miRNA seed) and the bin with the maximum CPDV selected.

### Metascape pathway analysis

In brief, the SCR-containing de-repressed gene lists derived from miR-302-SFI-treated cell lines, identified using the CPDV approach above, underwent pathway analysis using Metascape software (https://metascape.org/) [46]. For global mRNA data from clinical samples, we conversely selected the 1134 downregulated (repressed) targets in malignant GCT cases (log_2_ fold change <–1), of which 362 (31.9%) contained the SCR to the 2-7nt AAGUGC seed. These 362 downregulated genes were then analysed using Metascape.

### Flow cytometry

For cell cycle analysis, cells were analysed at d1-d4 post-transfection using Click-iT-EdU-Alexa-Fluor-647 Flow Cytometry Assay Kit (ThermoFisher Scientific). In brief, cells were incubated for up to 3 h with 10 µM of 5-ethynyl-2-deoxyuridine (EdU) dye. Cells (1 × 10^6^) were then collected and treated as per the manufacturer’s instructions. TCam-2 (Sem) and GCT44 (YST) cells were utilised for this work as 1411H (YST) cells did not adequately incorporate EdU dye. One µl of FxCycle Violet (ThermoFisher Scientific) was then added to the final mixture to stain cellular DNA and samples were analysed using a flow cytometer BD-LSR-Fortessa machine (BD Biosciences) at the Wellcome-MRC Cambridge Stem Cell Institute. For the detection of EdU with Alexa-Fluor-647, azide 633/635 nm excitation with a red emission filter (660/20 nm) was used. Flow cytometer data were analysed using FlowJo (FlowJo LLC, Becton Dickinson) (version 10.5.0).

### Statistics

Statistical analyses were performed using GraphPad Prism 6 software (GraphPad Software, La Jolla, US). As the variance between comparison groups was similar, an unpaired, two-tailed Student’s *t*-test was used for analyses. *P* values <0.05 were considered statistically significant unless otherwise stated. Data presented are mean values ± standard error of the mean.

## Results

### High overall 2-7nt AAGUGC seed abundance is universally present in malignant GCT clinical samples and cell lines

**Fig. 1:**
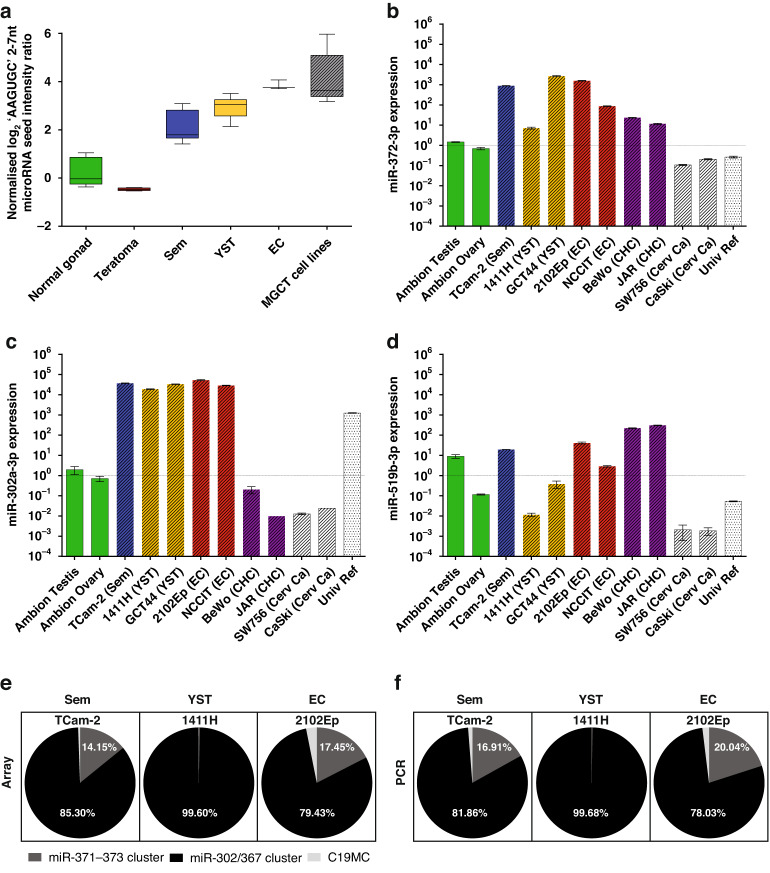
High abundance of miRNAs containing the key seed region AAGUGC in malignant GCT clinical samples and cell lines. **a** AAGUGC seed intensity in clinical samples and malignant GCT cell lines. Boxplot of summated miRNA microarray normalised log_2_ intensity ratios for the 12 miRNAs on the array containing the 2-7nt seed region AAGUGC. Shown are clinical samples, namely normal gonadal controls (*n* = 8, green), teratomas (*n* = 5, brown), seminoma (Sem) (*n* = 13, blue), yolk sac tumour (YST) (*n* = 12, yellow), embryonal carcinoma (EC) (*n* = 3, red) and malignant GCT (MGCT) cell lines (*n* = 6, grey hatched). **b**–**d** Relative qRT-PCR expression of **b** miR-372-3p, **c** miR-302a-3p, and **d** miR-519b-3p in testicular and ovarian controls (green), malignant GCT cell lines [blue = Sem, yellow = YST, red = EC, purple = choriocarcinoma (CHC); all hatched], non-GCT cell lines (white hatched), and Universal Reference (Univ Ref) RNA (white dotted—composed of equal quantities of total RNA from 10 human cell lines, including one EC cell line, hence some intermediate expression). **e**, **f** Relative contribution of each of the three AAGUGC seed-containing miRNA clusters (miR-371~373, miR-302/367, and C19MC) to overall seed density in seminoma (Sem; left), yolk sac tumour (YST; centre) and embryonal carcinoma (EC; right) cell lines by miRNA microarray (**e**, left) and qRT-PCR (**f**, right). Dark grey = miR-371~373, black = miR-302/367, light grey = C19MC.

Microarray data analysis demonstrated that intensity ratios for miRNAs containing the 2-7nt sequence AAGUGC, corresponding to the seed region of miR-371~373, miR-302/367, and specific C19MC miRNAs, were substantially higher in malignant GCT samples and cell lines than in non-malignant control tissues, comprising gonadal and teratoma tissues (Fig. 1a). Of note, the AAGUGC seed intensity for the non-seminomatous GCT subtypes YST and EC was higher than that observed for Sem samples (Fig. 1a). Malignant GCT cell lines had the highest overall 2-7nt AAGUGC seed abundance (Fig. 1a and Supplementary Fig. S1), highlighting their suitability for subsequent downstream functional assays. Intensity ratios for 10 miRNAs from these three malignant GCT-associated clusters, for each individual clinical sample (malignant GCTs and non-malignant controls) and cell lines, are shown in Supplementary Fig. S2. High levels of miRNAs contributing to the overall AAGUGC seed abundance in Sem, YST, and EC cell lines were next confirmed by qRT-PCR, compared with other cell lines and normal gonadal control samples (Supplementary Fig. S3), as exemplified by the representative miR-372-3p from the miR-371~373 cluster (Fig. 1b) and miR-302a-3p from the miR-302/367 cluster (Fig. 1c). We observed that although the very rare malignant GCT subtype CHC displayed high levels of miR-371~373 miRNAs, it demonstrated lower levels of miR-302/367 miRNAs and higher levels of C19MC miRNAs (miR-519b-3p; Fig. 1d), compared with the other common malignant GCT cell line subtypes Sem, YST, and EC. Whilst these cell line data are consistent with the presence of highly elevated levels of circulating miRNAs from the miR-371~373 cluster and C19MC at the time of CHC diagnosis in patient serum [20], it should be noted that these CHC cell lines were derived from placental CHC. In the absence of available GCT-derived CHC lines, these CHC cell lines were therefore not pursued further for in vitro analyses.

### MiR-302/367 cluster miRNAs individually make the largest relative contribution to high 2-7nt AAGUGC seed abundance in malignant GCTs

From the array intensity ratios, miRNAs from the miR-302/367 cluster made the largest overall contribution to AAGUGC seed abundance in malignant GCT cell lines, namely TCam-2 (Sem; 85.30%), 1411H (YST; 99.60%), and 2102Ep (EC; 79.43%) (Fig. 1e and Supplementary Table S5). Importantly, almost identical findings were confirmed by qRT-PCR (Fig. 1f and Supplementary Table S5), highlighting their appropriateness for further experimental study. Together, these data supported primarily targeting miR-302/367 cluster miRNAs, specifically miR-302a-d-3p (all containing the 2-7nt seed AAGUGC), in subsequent functional work in malignant GCT cells.

### Expression of miRNAs from the miR-371~373 and miR-302/367 clusters in malignant GCTs is positively correlated

**Fig. 2:**
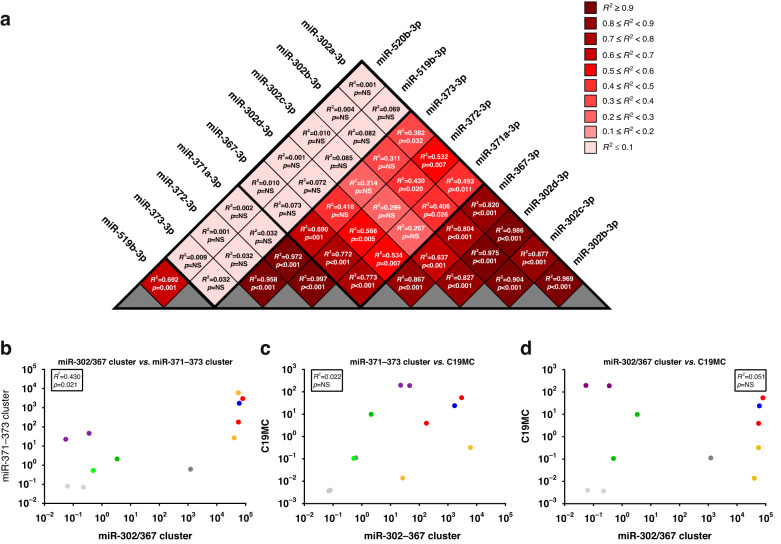
Correlation between expression levels of representative miRNAs within and across the miRNA clusters miR-371~373, miR-302/367, and C19MC. **a** Triangle plot showing the *R*^2^ and *p* values from linear regression analysis of individual qRT-PCR miRNA expression data from malignant GCT and non-GCT cell lines, Universal Reference RNA, and gonadal controls within and across the clusters miR-371~373, miR-302/267, and C19MC. Colour coding as per the *R*^2^ value key. **b**–**d** Comparison of aggregated qRT-PCR miRNA expression within a miRNA cluster versus other clusters. Aggregated expression from **b** miR-302/367 cluster (*n* = 5 miRNAs) vs. miR-371~373 cluster (*n* = 3), **c** miR-371~373 cluster vs. C19MC (*n* = 2), and **d** miR-302/367 cluster vs. C19MC. Colour coding: blue = SEM, yellow = YST, red = EC, purple = CHC, green = ovary/testes, light grey = non-GCT cell lines, dark grey = Universal Reference RNA. *R*^2^ and *p* values are shown for each comparison.

Linear regression analysis of miRNA qRT-PCR data showed a very strong positive correlation of expression levels for miRNAs within the two miRNA clusters, which are universally found at high levels in malignant GCTs (Sem, YST, and EC), namely miR-371~373 and miR-302/367 (Fig. 2a). *R*^2^ values for miRNAs from the miR-371~373 and miR-302/367 clusters, respectively, were 0.958–0.997 and 0.804–0.969 (*p* < 0.001 for all comparisons), with one exception (miR-302d-3p vs. miR-367-3p, *R*^2^ = 0.637, *p* = 0.002) (Fig. 2a). Next, miRNA expression was aggregated from the miR-371~373 cluster (*n* = 3 miRNAs), miR-302/367 cluster (*n* = 5), and C19MC (*n* = 2) to provide overall inter-cluster graphical comparisons (Fig. 2b–d). This was only significant for the miR-371~373 vs. miR-302/367 cluster comparison (*R*^2^ = 0.43, *p* = 0.021) (Fig. 2b), but not for miR-371~373 vs. C19MC nor miR-302/367 cluster vs. C19MC (Fig. 2c, d), as expected given high expression of C19MC only in the CHC malignant GCT subtype [14]. These highly significant positive intra-cluster and inter-cluster correlations for miR-371~373 and miR-302/367 suggested a potential single co-regulatory mechanism in malignant GCTs (Supplementary Fig. S4 and Supplementary Results/Discussion).

### Targeting the miR-371~373 and miR-302/367 clusters in malignant GCT cells

It was not possible to reliably target these two highly over-expressed miRNA clusters in malignant GCTs using CRISPR-Cas9 targeting at a genomic level, ‘gapmer’ inhibition of pri-miR-302/367, and PNA inhibition of mature miR-302/367 miRNAs (miR-302a-d). In short, CRISPR/Cas9 was challenging for multiple reasons. First, optimal design/selection of CRISPR RNAs (crRNAs, i.e., guide RNAs) was challenging because it was difficult to accurately identify the precise promoter location and transcriptional start sites for miRNAs [47]. Second, the selected crRNA guides proved inefficient in excising the relevant regions of DNA. Third, as a consequence of low excision efficiency (2-12%; Supplementary Table S2), an additional process was subsequently required to select individually excised cells, but unfortunately, these were not viable for long-term growth. We next attempted miRNA depletion testing multiple gapmers individually against the pri-miR-302/367 sequence, but at best, only modest depletion of mature miR-302/367 miRNA expression was obtained (15.4–26.7%), and this was not associated with any observed reduction in cell numbers (data not shown). Finally, with PNA inhibition, after initial success, a lack of reproducibility of results was observed due to batch-to-batch variation; after further enquiry with the manufacturer, they confirmed they had altered the proprietary chemistry of the PNA, which meant that this inhibition approach was not pursued further (data not shown).

### Mature miRNA targeting approach: miR-302-SFI LNA-based targeting of miR-302a-d-3p miRNAs

**Fig. 3:**
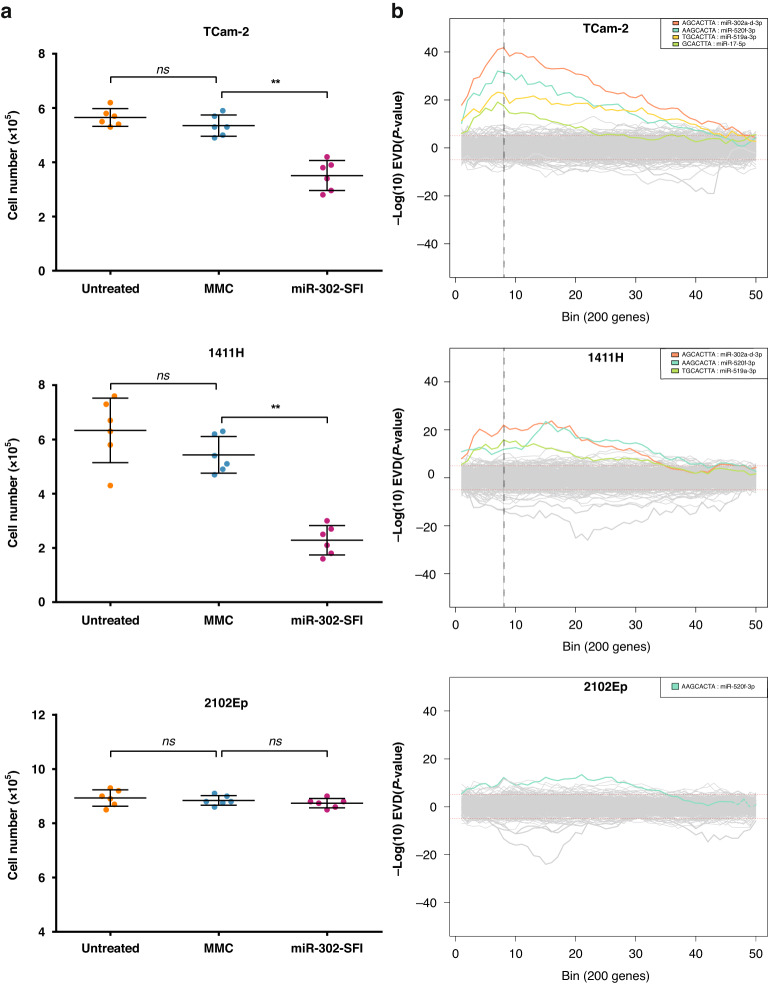
Functional phenotypic and genotypic effects of the miR-302 super-family-inhibitor (miR-302-SFI) in malignant GCT cells. **a** Cell numbers for TCam-2 (Sem; upper panel), 1411H (YST; central panel), and 2102Ep (EC; lower panel) at day 7 in untreated cells, mismatched control (MMC), and miR-302-SFI-treated cells. Key: **p < 0.01; ns = not significant. **b** *Sylamer* landscape plots for TCam-2 (upper panel), 1411H (central panel), and 2102Ep (lower panel) cells treated with miR-302-SFI, compared with MMC, at day 2 post-transfection. Log_10_ transformed, sign-adjusted enrichment *p* values for each seed complementary region (SCR) ‘word’ relative to all other words are plotted on the *y*-axis against the ranked gene list on the *x*-axis, which has upregulated (de-repressed) genes to the left and downregulated genes to the right. SCRs corresponding to true miRNAs (as listed in miRBase version 22.1) that reach significance by extreme value distribution and which correspond to the AAGUGC seed sequence are shown in colour; their *p* values are listed in the top right of the graph. Non-significant word plots are shown in grey. The change point detection value (CPDV) algorithm was used to determine the most appropriate *Sylamer* enrichment peak for selecting gene lists for further analyses. The bin with the maximum CPDV was selected and indicated by the vertical dotted line; this represented bin 8 (1600 genes) for both the TCam-2 and 1411H cell lines.

Following miR-302-SFI transfection in three representative malignant GCT cell lines, cell growth was assessed by cell counts (Fig. 3a). Importantly, no difference was observed between untreated and control (MMC) treated groups. In TCam-2 (Sem) and 1411H (YST) cell lines, a reduction in cell numbers was observed at d7 in the miR-302-SFI-treated group (Fig. 3a, upper/central panels, respectively), but not in 2102Ep (EC) cells (Fig. 3a, lower panel). In all experimental conditions (miR-302-SFI-treated, MMC-treated, and untreated cells), live cells ranged from 93.0% to 96.3% of the total cell population counted (Supplementary Table S6). It was therefore concluded that the reduction in cell numbers seen was due to reduced cell proliferation rate rather than due to apoptosis/cell death. Subsequent study therefore sought to explore in more detail the reduced cell proliferation rate observed following miR-302-SFI treatment through genotypic/pathway analyses.

### Global mRNA microarray expression profiling of miR-302-SFI-treated cells

Profiling was undertaken at 48 h post-transfection for miR-302-SFI-treated cells compared with MMC-treated and untreated cells, and data used to produce *Sylamer* landscape plots (Fig. 3b). In the TCam-2 plot, the most significant peak was that for the key SCR (GCACTT) corresponding to the shared 2-7nt AAGUGC seed of miR-302a-d-3p (Fig. 3b, upper panel). In addition, significant signals were obtained for other miRNAs that contain the AAGUGC seed, namely miR-520f-3p (3-8nt), miR-519a-3p (2-7nt) (both C19MC miRNAs), and miR-17-5p (1-6nt; from the oncogenic miR-17~92 cluster) (Fig. 3b, upper panel). For 1411H, the miR-302a-d-3p peak was again the most significant (Fig. 3b, central panel), and the other significant peaks corresponded to C19MC miRNAs miR-520f-3p and miR-519a-3p. In the 2102Ep (EC) plot (Fig. 3b, lower panel), there were no significantly enriched signals for the core SCR GCACTT corresponding to the 2-7nt AAGUGC seed, consistent with the lack of phenotype observed on d7. Only a weak signal, corresponding to the single C19MC miRNA miR-520f-3p (3-8nt AAGUGC), was seen. Accordingly, only seminoma and YST cells were taken forward for further downstream analyses.

### Identification of de-repressed genes following miR-302-SFI transfection using *Sylamer* assessment of global mRNA expression data

Using the CPDV algorithm, bin 8, corresponding to the first 1600 de-repressed genes on the left of the *x*-axis, was identified as the optimal *Sylamer* cut-off for both TCam-2 and 1411H cells (Fig. 3b, upper/central panels). For TCam-2, of these 1600 genes, 881 (55.1%) were found to have one or more SCRs for AAGUGC in their 3’UTRs. As 16% of all human mRNAs contain the SCR GCACTT corresponding to the 2-7nt AAGUGC seed in their 3’UTRs [14], this represented significant enrichment of the SCR in upregulated (de-repressed) genes following miR-302-SFI treatment (*χ*^2^ statistic 57.6, *p* < 0.01). Similarly, for 1411H, 775 genes (48.4%) out of 1600 contained the SCR of interest (*χ*^2^ statistic 39.8, *p* < 0.01). Of note, 188 SCR-containing mRNA targets (24.2% overlap) were common to both TCam-2 and 1411H (Supplementary Table S7).

### Metascape pathway analysis and clinical correlation of malignant GCT cell line findings

**Fig. 4:**
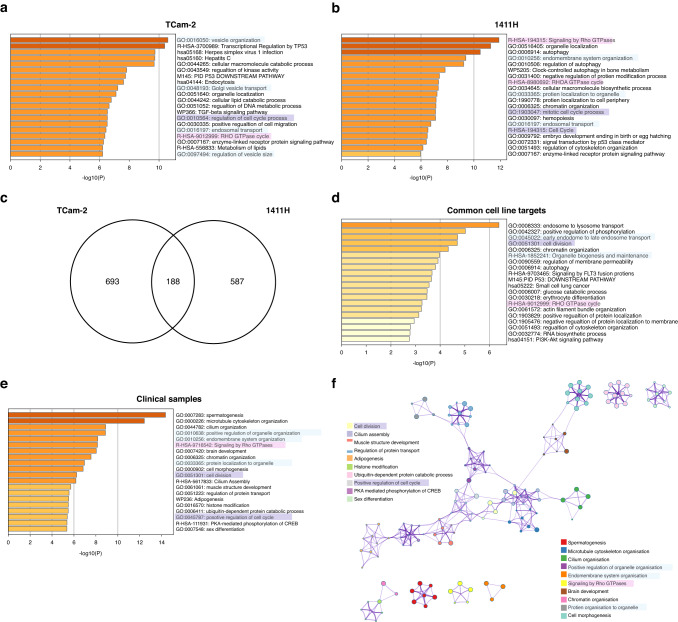
Metascape pathway analysis of miR-302-SFI effects in malignant GCT cells and corroboration in clinical samples. **a**–**d** Metascape pathway analysis was undertaken for de-repressed mRNAs containing the seed complementary region (SCR) GCACTT, complementary to the 2-7nt AAGUGC miRNA seed of miR-302a-d-3p, and the top-ranking 20 pathways/terms listed in bar plots with significance values. **a** TCam-2 (Sem; *n* = 881 genes), **b** 1411H (YST; 775 genes), **c** Venn diagram showing there are 188 overlapping common de-repressed mRNA targets between TCam-2 and 1411H, **d** The common mRNA targets for the cell lines TCam-2 and 1411H, **e** Corroboration in malignant GCT clinical samples. Metascape pathway analysis for corresponding downregulated (repressed) genes (*n* = 362) derived from clinical malignant GCT cases. **f** Associated network analysis derived from data in (**e**). Key: colour-coding for the three recurring Metascape pathways, namely molecular signal transduction through Rho-GTPases (highlighted in pink), vesicle organisation/transport (light blue), and cell cycle regulation/division (purple). All related GO terms are highlighted, e.g., for vesicle organisation/transport, relevant GO pathways were identified by searching for ‘vesicle’ in the main and related ‘child’ GO terms, and highlighted accordingly.

The lists of SCR-containing, de-repressed genes in TCam-2 (*n* = 881) and 1411H (*n* = 775) next underwent pathway analysis using Metascape; multiple significant biological processes were identified (Fig. 4a, b, respectively). In particular, three cellular processes were recurrently involved through AAGUGC seed-containing miRNA targeting, namely molecular signal transduction via Rho-GTPases, vesicle organisation/transport, and cell cycle regulation/division (Fig. 4a, b). Next, we interrogated the 188 common targets to both TCam-2 and 1411H (Fig. 4c) and identified that this core set also contained these three key processes (Fig. 4d). Next, to establish the clinical relevance of these findings, we interrogated the corresponding downregulated (repressed) genes (*n* = 362) derived from clinical malignant GCT cases. Importantly, these three processes all featured prominently (Fig. 4e), demonstrated in the associated network analysis (Fig. 4f). Together, these data confirm the clinical relevance of our miR-302-SFI approach in malignant GCT cell lines.

### Cell cycle analysis

**Fig. 5:**
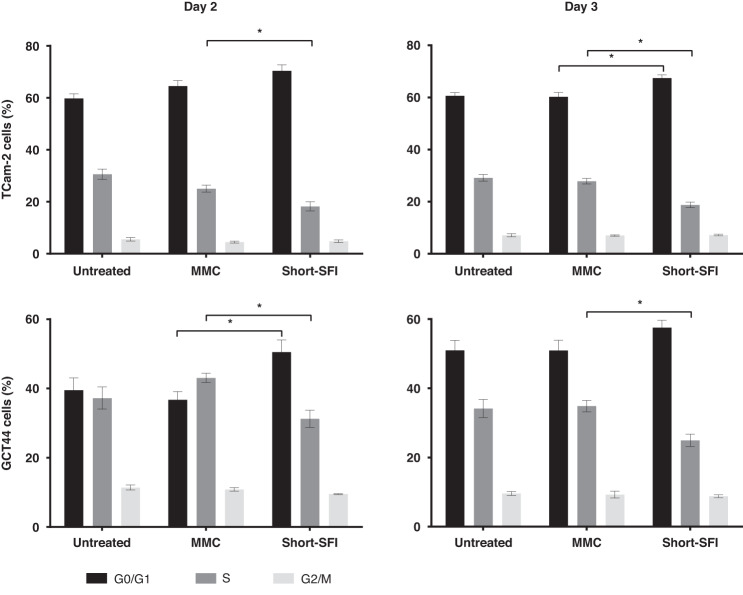
Effect of short-SFI treatment on cell cycle progression in malignant GCT cells. Cell cycle analysis following treatment of malignant GCT cells with the 10nt short-SFI. Summary data of the percentage (%) of live, single cells in G0/G1 (black), S (dark grey) and G2/M (light grey) phase from biological duplicate experiments of cell cycle analysis on TCam-2 (Sem; upper panel) and GCT44 (YST; lower panel) on day 2 (left) and day 3 (right) in untreated cells and cells post-transfection with the short-SFI or mismatched control (MMC).

Next, cell cycle regulation was assessed directly, due to growth inhibition (Fig. 3a) and affected cell cycle pathway regulation following miR-302-SFI treatment (Fig. 4). In initial optimisation experiments using TCam-2, use of the longer, standard miR-302-SFI resulted in subtle decreases in the proportion of cells in S-phase and concomitant increases in those in G0/G1-phase at d2, d3, and d4 compared with MMC-treated cells (Supplementary Table S8). However, the identification from *Sylamer* analysis of the presence of signals for other miRNAs such as miR-519a-3p (2-7nt AAGUGC) and miR-520f-3p (1-6nt AAGUGC) from C19MC following miR-302-SFI treatment (Fig. 3b) led to the hypothesis that inhibition, and thus effects on cellular processes, could be enhanced by use of a shorter inhibitor that could more widely target AAGUGC seed-containing miRNAs from miR-302/367, miR-371~373, and C19MC. Accordingly, cell cycle analysis was undertaken on 10nt ‘short-SFI’ treated TCam-2 (Sem) and GCT44 (YST) cells and compared with MMC-treated cells. Short-SFI treatment resulted in a decrease in the proportion of cells in S-phase and an increase in those in G0/G1-phase at d2 and d3 (Fig. 5 and Supplementary Table S9). Together, these data suggest that inhibition of 2-7nt AAGUGC seed-containing miRNAs results in increased cell cycle arrest at the G0/G1 checkpoint and reduced G1/S transition.

## Discussion

Here, we extend our previous observations demonstrating universal over-expression of the miR-371~373 and miR-302/367 clusters in all malignant GCTs, regardless of patient age, tumour site, and subtype, associated with global downregulation of mRNA targets [14]. We confirmed that the cell line panel was representative of miRNA changes in clinical malignant GCT tissues, and therefore suitable for use in experimental studies. However, the ability to demonstrate the functional significance of the over-expressed miR-371~373 and miR-302/367 clusters is challenging. First, six of the eight main miRNAs from these two clusters contain an identical 2-7nt AAGUGC seed region, which principally determines mRNA target binding [14], and furthermore, miR-371a-3p contains 1-6nt AAGUGC which also contributes to targeting [18]. Consequently, substantial redundancy exists, which needs to be overcome in order to reliably demonstrate a functional role for these miRNAs. A second challenge is that these six 2-7nt AAGUGC-containing miRNAs derive from two different genomic loci, namely chromosomes 19q13.41 (miR-371~373) and 4q25 (miR-302/367). Different experimental approaches were attempted to overcome this redundancy, including use of CRISPR-Cas9, gapmer, and PNA systems, which were not pursued further due to limitations (Supplementary Discussion).

Ultimately, we used an LNA/DNA combination miRNA inhibitor (miR-302-SFI) to demonstrate a negative growth phenotype in malignant GCT cells, resulting in de-repression of multiple mRNA targets. This inhibitor predominantly targeted miR-302a-d-3p from the miR-302/367 cluster, which made up the largest contribution to 2-7nt AAGUGC seed abundance in malignant GCT cells. Pathway analysis identified three key cellular processes that were recurrently affected by inhibition, namely Rho-GTPase signalling, vesicle organisation/transport, and cell cycle regulation, processes importantly corroborated in clinical samples. Of note, we previously identified miR-371~373 and miR-302/367 mediated intracellular signalling, including through GTPases, as fundamental in malignant GCT tissues [14]. In addition, miR-302-SFI treatment affected pathways involved in vesicle organisation and transport, critical in the production of extracellular vesicles (EVs) that allow miRNAs, particularly miR-371a-3p, to be released into the tumour microenvironment (TME) and then into the circulation, with biomarker potential. Importantly, malignant GCT cells communicate with non-tumour stromal cells of the TME through the release of EVs enriched in oncogenic miRNAs, with miR-371a-3p/-5p the most abundant, likely contributing to tumour progression [48]. Accordingly, miR-371a-3p/-5p effects on TME cells included increased collagen contraction in fibroblasts and angiogenesis in endothelial cells [48].

**Fig. 6:**
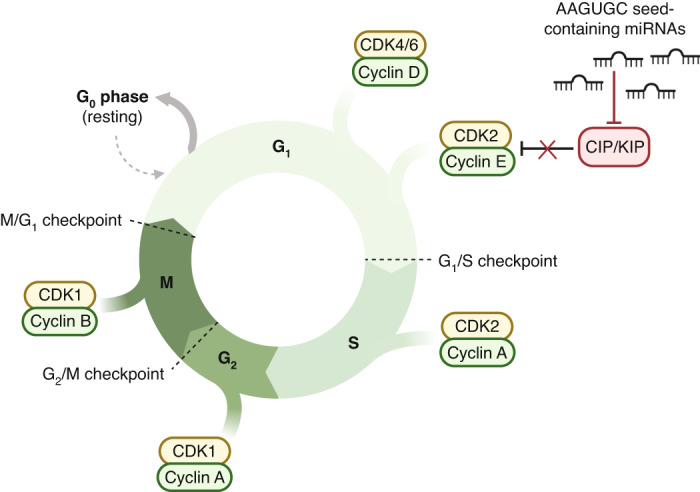
Graphical abstract summarising potential effects of AAGUGC seed-containing miRNAs on the cell cycle in malignant germ cell tumours (GCTs) and similar biological systems. Data from this manuscript and published studies support roles for AAGUGC seed-containing miRNAs in cell cycle regulation in malignant GCTs and embryonic stem cells [49–57]. These miRNAs include members from the miR-302/367, miR-371~373, C19MC, and miR-17~92 clusters. This regulation is likely mediated through inhibition of Cyclin-dependent kinase (CDK)-interacting protein/Kinase inhibitory protein (CIP/KIP) family members such as p21, p27, and p57, which are known targets of these miRNAs. Accordingly, high levels of AAGUGC-containing miRNAs result in low levels of CIP/KIP members, with concomitant increased Cyclin levels, allowing cell cycle progression. Conversely, as demonstrated in this study, inhibition of these upregulated miRNAs predominantly reduces cell cycle proliferation likely mediated through effects on G0/G1 and G1/S transition. Schematic created with BioRender.com.

MiR-302-SFI effects on malignant GCT cells were mediated at least in part through cell cycle disruption, in particular G0/G1 and G1/S transition, consistent with miR-302/367 inhibition in human embryonic stem cells (ESCs), where modest changes in individual expression of multiple genes were also observed [49, 50], as expected in miRNA perturbation experiments [16]. Indeed, in *Dicer1* knockout human ESCs, re-introduction of individual 2-7nt AAGUGC seed-containing miRNAs from the miR-371~373 and miR-302/367 clusters alone was sufficient to promote cell growth and survival [51], in addition to similar findings in murine ESCs [52–54] via regulation of G1/S transition. In addition, the oncogenic miR-17~92 cluster (known as OncomiR1) has been extensively studied, and many miRNA members of this cluster, through the common AAGUGC seed, share mRNA targets with miR-302a-d-3p. Indeed, following miR-302-SFI treatment, our data showed enrichment in upregulated mRNAs for miR-17-5p (3-8nt AAGUGC) from the miR-17~92 cluster in TCam-2 cells (Fig. 3b). Furthermore, experimentally proven effects of miR-17~92 miRNAs include the promotion of proliferation and maintenance of cell survival through repression of key cell cycle associated targets such as *P21*, *BIM*, *PTEN* and *CTGF* [55, 56]. Of note, miR-302/367 miRNAs also directly contribute to the regulation of *P21* expression in human ESCs [57]. Taken together, these data from similar systems reinforce our findings here for malignant GCTs that AAGUGC seed-containing miRNAs alter cell cycle regulation in G1/S transition, summarised in Fig. 6. Inhibiting these key AAGUGC seed-containing miRNAs in malignant GCTs, which recapitulate the ESC-associated miRNA environment, therefore represents a rational therapeutic strategy. Moreover, cisplatin resistance in malignant GCTs is mediated through further over-expression of miR-371~373 miRNAs [21] (and thus cellular AAGUGC seed abundance levels), lending further weight to this approach.

This work represents an important foundation for future studies. As there is minimal/no expression of miR-371~373 nor miR-302/367 miRNAs in normal, differentiated tissues [14], we anticipate minimal/no unexpected inhibitor off-target effects in vivo [1]. An inhibitor could be used clinically as monotherapy or in combination with reduced-dose platinum-based chemotherapy, to improve outcomes, of relevance as GCTs affect a predominantly young population [12]. Importantly, miRNA therapeutics are already in clinical use. For example, the 15nt LNA miR-122 inhibitor miravirsen has completed Phase-II trials in hepatitis-C patients, showing sustained and dose-dependent decreases in viral load [58] with no long-term safety issues [59]. A recent phase-I trial of miR-16 replenishment for mesothelioma patients [60] further demonstrates their potential clinical use [61].

Our current study has a number of limitations. First, we used an LNA/DNA mixmer containing the maximal LNA content possible (70%), as per Santaris Pharma intellectual property rights, with effects in Sem/YST but not EC cells. A fully 100% LNA inhibitor, with an even higher affinity for target miRNAs, could potentially yield greater inhibition and therefore wider and more substantial cellular effects, but we were unable to test this. We acknowledge that further work is now necessary and warranted in malignant GCTs, including in further EC cell lines and in vivo models, and combinatorial treatments using miRNA inhibition with platinum agents such as cisplatin, outside the remit of this study. In summary, we demonstrate the functional significance of miR-371~373 and miR-302/367 over-expression in malignant GCTs in vitro. This serves as a platform for further work, with the ultimate aim of improving clinical outcomes.

### Supplementary information

Supplementary Information

Supplementary Figure S1

Supplementary Figure S2

Supplementary Figure S3

Supplementary Figure S4

Supplementary Tables S1–S9

## Data Availability

Data that support the findings of this study are available from the corresponding authors upon request.
