# Correction: Targeting oncogenic microRNAs from the miR-371~373 and miR-302/367 clusters in malignant germ cell tumours causes growth inhibition through cell cycle disruption

**DOI:** 10.1038/s41416-023-02479-5

**Published:** 2023-11-02

**Authors:** Shivani Bailey, Marta Ferraresso, Luz Alonso-Crisostomo, Dawn Ward, Stephen Smith, James C. Nicholson, Harpreet Saini, Anton J. Enright, Cinzia G. Scarpini, Nicholas Coleman, Matthew J. Murray

**Affiliations:** 1 Department of Pathology, University of Cambridge, Cambridge, CB2 1QP UK; 2 Department of Paediatric Haematology and Oncology, Cambridge University Hospitals NHS Foundation Trust, Cambridge, CB2 0QQ UK; 3 Department of Paediatrics, University of Cambridge, Cambridge University Hospitals NHS Foundation Trust, Cambridge, CB2 0QQ UK; 4 EMBL-European Bioinformatics Institute (EMBL-EBI), Wellcome Genome Campus, Hinxton, Cambridge, CB10 1SD UK; 5 Department of Histopathology, Cambridge University Hospitals NHS Foundation Trust, Cambridge, CB2 0QQ UK

**Keywords:** Germ cell tumours, miRNAs

Correction to: *British Journal of Cancer* 10.1038/s41416-023-02453-1, published online 03 October 2023.

In this article, the original title ‘Targeting oncogenic microRNAs from the miR-371 ~ 373 and miR-302/267 clusters in malignant germ cell tumours causes growth inhibition through cell cycle disruption’ contains a typographical error.

It should read:

Targeting oncogenic microRNAs from the miR-371 ~ 373 and miR-302/367 clusters in malignant germ cell tumours causes growth inhibition through cell cycle disruption

The original article has been corrected.

