# Supplementary material for: Targeting oncogenic microRNAs from the miR-371~373 and miR-302/367 clusters in malignant germ cell tumours causes growth inhibition through cell cycle disruption
Source: Br J Cancer. 2023 Oct 3;129(9):1451–61. doi: 10.1038/s41416-023-02453-1 (PMC10628203; doi:10.1038/s41416-023-02453-1)
Supplement: Supplementary file 1 — Supplementary Information [file 41416_2023_2453_MOESM1_ESM.docx]

**Supplementary Information**

**Supplementary Methods**

**CRISPR-Cas9 targeting of the miR-371~373 and miR-302/367 clusters.** The clustered regularly interspaced palindromic repeats (CRISPR)-CRISPR-associated nuclease 9 (Cas9) system was utilised for this work, using a DNA plasmid-free, Cas9 protein-based transfection system (Dharmacon). CRISPR-RNAs (crRNAs, i.e., guide RNAs) were designed to flank the genomic loci of the miR-371~373 and miR-302/367 clusters using the Dharmacon online crRNA design tool (Dharmacon, Horizon Discovery Group, Lafayette, CO, USA). Following screening of eight candidates for predicted cutting efficiency, two crRNAs were selected for each cutting site for *in vitro* testing (Supplementary-Table-S2). These crRNA sequences were transfected into GCT44 cells, seeded at 1.4x10^4^ cells in 96-well plates. In addition, crRNA and Cas9 protein was transfected into cells using the DharmaFECT Duo system, as per the manufacturer’s instructions (Dharmacon). The crRNA guides the Cas9 nuclease, resulting in a specific double stranded break, providing the sequence lies immediately adjacent to a protospacer adjacent motif (PAM) three nt sequence (namely ‘NGG’, where ‘N’ represents any nucleotide). Genomic DNA was subsequently extracted, purified using the QIAquick PCR purification kit (Qiagen), and mismatch detection assays undertaken to determine the cutting efficiency of the crRNAs according to published protocols (1, 2).

**Gapmer inhibition of miR-302/367 miRNAs (miR-302a-d). ‘**Gapmers’ are LNA- and DNA-based single-stranded antisense oligonucleotides designed to target long non-coding RNAs (lncRNAs), mRNAs, and miRNAs (3). The sequence 300bp either side of the miR-302/367 cluster pri-miRNA was submitted to Exiqon’s LNA team. Five gapmer sequences were designed targeting various loci within the pri-miR-302/367 sequence, ensuring that the hairpin loops of the miRNAs within this pri-miRNA sequence were excluded. (Supplementary-Table-S3). A positive control gapmer inhibitor *versus* the lncRNA MALAT1 (4) (Gene ID: ENSG00000251562, Exiqon Product Number 300630-101, proprietary sequence) was utilised along with an appropriate mismatch control (MMC) gapmer (300610) as a negative control, to confirm efficacy of the transfection reagent Viromer Blue (Lipocalyx GmbH, Halle, Germany). Following optimisation, experiments were performed in 6-well plates, with cells seeded to reach ~40% confluence at transfection on d0, at a final gapmer concentration of 37nM for all cell lines tested (TCam-2, GCT44, 1411H, and 2102Ep). The media containing the transfection reagent Viromer Blue (1µl per 500µl transfection solution) was changed five hours (5h) post-transfection for fresh media. Cells were cultured for 48h following transfection and then total RNA extracted using TriReagent (Sigma-Aldrich), following the TriZol RNA extraction protocol (5) and utilised for subsequent miRNA and lncRNA quantification.

**Peptide nucleic acid (PNA) inhibition of miR-302/367 miRNAs (miR-302a-d).** PNAs are composed of N-(2-aminoethyl)-glycine units linked by peptide bonds; the nucleobases are attached to this backbone via a methylene carbonyl linkage, rendering the PNA charge-neutral and thus facilitating uptake into cells (6). An 18nt-long PNA-inhibitor targeting the functional unit miR-302a-d-3p [i.e., the four miRNAs miR-302a-3p, miR-302b-3p, miR-302c-3p, and miR-302d-3p, all of which have very similar nucleotide sequences and contain the 2-7nt AAGUGC seed (7)] was designed with the assistance of Dr Mike Gait (Laboratory of Molecular Biology, Cambridge, UK) and modified to include a cell-penetration-peptide (CPP) to aid cellular entry, and acetylation at the N-terminus of the Tat peptide to increase stability. A PNA-mismatched-control (PNA-MMC) comprised identical nucleotides and length, but with scrambled sequence (Supplementary-Table-S4), and was screened to confirm that it had no bioinformatically-predicted miRNA targets. The PNA-MMC was used to confirm specificity of results obtained with the PNA-inhibitor. Experiments were performed in 24-well plates in biological triplicate using cell lines TCam-2, 1411H, GCT44, and 2102Ep. Cells were seeded on day -1 (d-1) to allow ~40% confluence at transfection on d0. On d0, PNA-inhibitor and PNA-MMC were activated through heat treatment at 70°C for 5 minutes and immediately transferred to ice, as per the manufacturer’s instructions (Panagene, Daejeon, Korea). Culture media was then aspirated from the plate wells and 400μl of media containing either PNA-MMC or PNA-inhibitor was added directly at 1µM concentration. Plates were incubated and media replaced every 24h for the duration of the experiment. Cells were counted at each time-point (d4/d7) using a Countess automated-cell-counter (Invitrogen), as described (8), prior to lysis and RNA extraction using the PureLink miRNA isolation kit (Invitrogen, ThermoFisher Scientific), as per the manufacturer’s instructions.

**Supplementary Results**

**MiR-371~373 and miR-302/367 over-expression in malignant GCTs is not due to genomic gain or amplification.** To explore whether copy number change was the regulatory mechanism underpinning miR-371~373 and miR-302/367 over-expression observed in malignant GCTs, quantification of genomic content for upstream and downstream loci at the two regions encoding these clusters (chromosome 19q13.41 and 4q25, respectively) was performed in malignant GCT cell lines. However, no consistent gain or amplification was observed at either loci (Supplementary-Figure-S4), suggesting that the miR-371~373 and miR-302/367 cluster over-expression seen in malignant GCTs is likely due to an alternative mechanism.

**Targeting the miR-371~373 and miR-302/367 clusters in malignant GCT cells.** A number of strategies were employed to target miRNAs from these clusters at the genomic, transcriptional, and mature miRNA level, outlined below.

**Genomic-level approach: CRISPR-Cas9 targeting of the miR-371~373 and miR-302-367 clusters**. A CRISPR-Cas9 system was utilised in an attempt to target the miR-371~373 and miR-302/367 clusters using two sets of double-stranded breaks on either side of each cluster. However, the crRNAs that were designed and tested lacked adequate cutting efficiencies (Supplementary-Table-S2) to proceed further with this work and accordingly, other methods of miRNA targeting were pursued.

**Primary transcript-level approach: gapmer targeting of the pri-miRNA miR-302/367 transcript.** Due to lack of efficacy with the CRISPR-Cas9 genomic approach, and as the greatest contribution to 2-7nt AAGUGC miRNA seed abundance in malignant GCTs is from the miR-302/367 cluster, we next explored targeting this miRNA cluster alone at the pri-miRNA level. Overall, there was no substantial decrease in miR-302/367 expression following transfection with the five gapmers against the miR-302/367 pri-miRNA sequence that were designed and tested. The three gapmers with the lowest miR-302/367 miRNA expression levels (miR-302c-3p relative expression of 84.6%, 75.2%, and 73.3%) following transfection were subsequently assessed in cell growth assays. However, there was also no significant difference in TCam-2 (Sem) cell numbers at d7 following transfection with any of these three gapmers tested (data not shown). These data confirmed the substantial functional redundancy for 2-7nt AAGUGC seed containing miRNAs in malignant GCTs (7) and suggested a more complete inhibition and/or different approach would be required to obtain phenotypic consequences.

**Mature transcript-level approach: CPP-PNA targeting of mature miR-302a-d family miRNAs.** Next, we sought to target the 2-7nt AAGUGC-containing mature miR-302a-d miRNA family in malignant GCTs, namely miR-302a-3p, miR-302b-3p, miR-302c-3p, and miR-302d-3p. In initial experiments, CPP-PNA inhibition reduced mature miR-302a-d levels and limited cell growth compared with MMC treated cells (data not shown). However, subsequent variation was observed in the subsequent results obtained, depending on manufacturer batch number. This lack of reproducibility due to batch-to-batch variation meant that further use of CPP-PNA inhibitors was not indicated. Accordingly, the identical 16nt sequence designed for CPP-PNAs was used for our next studies using LNA-DNA mixmer based inhibition.

**Supplementary Discussion**

We were not able to reliably target the highly overexpressed miR-371~373 and miR-302/367 clusters in malignant GCTs using CRISPR-Cas9 targeting at a genomic level, ‘gapmer’ inhibition of pri-miR-302/367, and peptide nucleic acid (PNA) inhibition of mature miR-302/367 miRNAs (miR-302a-d-3p). The genomic approach using CRISPR-Cas9 was limited firstly by the difficulties associated with identifying the promoters and transcriptional start sites of miRNAs (9). Secondly, making two equally efficacious double-stranded cuts on either side of the genomic region coding for the primary miRNA transcripts (pri-miRNAs) proved difficult. Only a low crRNA cutting efficiency was obtained, meaning that cells then needed to be sorted to select out those which had been successfully excised, from the majority of cells in whom the two genomic loci remained intact. However, the subsequently selected excised malignant GCT cells did not grow in single cell culture and could not therefore be used for this approach.

The use of a panel of gapmer inhibitors to target the primary miRNA transcript (pri-miRNA) of the miR-302/367 cluster (the cluster making the largest contribution to 2-7nt AAGUGC seed abundance in malignant GCT cells) also did not result in significant reductions in the expression of the mature miRNAs of this cluster in our studies. Designing and trialling further gapmer inhibitor sequences may yield one that results in more successful pri-miRNA degradation. However, having designed and tested five gapmers, with a maximal reduction in mature miRNA levels of <30%, and given the known substantial functional redundancy in this system, we did not pursue this approach further at this time.

Finally, with PNA inhibition, after initial success in terms of a reduction in cell numbers, a lack of reproducibility of results was observed due to batch-to-batch variation. In further discussion with the manufacturer, they confirmed they had altered the proprietary chemistry of the PNA in subsequent batches, with which we could not obtain reliable results and replicate our original findings. Despite requests, the manufacturer would not return to the original proprietary PNA chemistry to allow us to pursue this approach further, and hence it was abandoned.

**Legends to Supplementary Figures and Tables**

**Supplementary-Figure-S1. High abundance of miRNAs containing the key seed region AAGUGC in malignant germ cell tumour (GCT) clinical samples and cell lines.** AAGUGC seed density in clinical samples and individual GCT cell lines. Boxplot of summated miRNA microarray normalised log_2_ intensity ratios for the 12 miRNAs on the array containing the 2-7nt seed region AAGUGC. Shown are clinical samples (for comparison purposes; identical to Figure-1A), namely normal gonadal controls (n=8, green), teratomas [n=5, brown; both mature (MT) and immature (IT)], seminoma (Sem) (n=13, blue), yolk sac tumour (YST) (n=12, yellow), and embryonal carcinoma (EC) (n=3, red). Next are the data for the six GCT cell lines, colour-coded as above for the clinical samples. Note that two of the six GCT cell lines, namely PA1 and Tera-2, typically have a major teratoma component, and thus display intermediate miRNA intensity.

**Supplementary-Figure-S2. Microarray normalised log_2_ intensity ratios for ten individual miRNAs from the miRNA clusters miR-371~373 (n=5), miR-302/367 (n=3), and C19MC (n=2) in individual clinical samples and cell lines.** Intensity plots are shown for: A) miR-371a-3p, B) miR-372-3p, C) miR-373-3p, D) miR-302a-3p, E) miR-302b-3p, F) miR-302c-3p, G) miR-302d-3p, H) miR-367-3p, I) miR-519b-3p, and J) miR-520b-3p. Colour coding: normal gonadal controls (green), teratomas (brown), seminoma (blue), yolk sac tumour (yellow), and embryonal carcinoma (red), and malignant GCT cell lines (n=6, grey hatched). Ratios are expressed relative to the mean intensity in the non-malignant clinical samples, namely gonadal controls and teratomas, which together have an intensity ratio of 0. Note that two of the six GCT cell lines, namely PA1 and Tera-2 (representing the 1^st^ and 3^rd^ columns) typically have a major teratoma component, and thus display intermediate miRNA expression.

**Supplementary-Figure-S3. Quantitative PCR (qRT-PCR) confirmation of miRNA expression levels in cell lines.** Relative qRT-PCR expression level plots for individual miRNAs are shown for: A) miR-371a-3p, B) miR-372-3p, C) miR-373-3p, D) miR-302a-3p, E) miR-302b-3p, F) miR-302c-3p, G) miR-302d-3p, H) miR-367-3p, I) miR-519b-3p, and J) miR-520b-3p. Colour coding: gonadal controls (green), seminoma (Sem; blue hatched), yolk sac tumour (YST; yellow hatched), embryonal carcinoma (EC; red hatched), choriocarcinoma (CHC; purple hatched), cervical carcinoma (white hatched), Universal Reference (white dotted - composed of equal quantities of total RNA from 10 human cell lines, including one EC cell line, hence some intermediate expression). The black horizontal line at 10^0^ (i.e., 1) represents the mean expression from the Ambion Ovary and Ambion Testis samples, as a reference value.

**Supplementary-Figure-S4. Genomic copy number determination for upstream and downstream miR-371~373 and miR-302/367 regions.** Copy number determined by quantitative PCR, normalised to four housekeeping genes, and compared with human testis genomic DNA (gDNA). Data for four representative malignant GCT cell lines for the A) miR-371~373 cluster and B) miR-302-367 cluster. Colour coding as in Supplementary-Figure-S1. Malignant GCT cell lines are represented by hatched columns.

**Supplementary-Table-S1. Primer sequences for genomic copy number assessment.** Forward and reverse primer sequences of miRNA clusters of interest and housekeeper genes used for genomic copy number assessment in malignant GCT cell lines and normal testis DNA. Primers were designed using the website ‘Primer3’ (<https://bioinfo.ut.ee/primer3/>) and obtained from Sigma-Aldrich.

**Supplementary-Table-S2.** **CRISPR-Cas9 RNA (crRNA) sequences targeting miR-371~373 and miR-302/367 clusters at a genomic level.** Sequences and positions of CRISPR-Cas9 RNAs (crRNAs) targeting the miR-371~373 and miR-302/367 clusters are shown. Cutting efficiencies (%) of the CRISPR-Cas9 RNA (crRNA) sequences targeting miR-371~373 and miR-302/367 clusters at a genomic level. Percentages were calculated based on densitometry assessment of gel images.

**Supplementary-Table-S3.** **Gapmer inhibitor sequences targeting miR-302/367 miRNAs at a primary transcript level (pri-miR-302/367).** Sequences of gapmers designed to target the miR-302/367 cluster and sequence of the negative control. The sequence of the positive control MALAT1 gapmer is proprietary and protected by copyright (Exiqon/Qiagen, product number 300630-101).

**Supplementary-Table-S4. Peptide nucleic acid** (**PNA) inhibitor sequences targeting miR-371~373 and miR-302/367 cluster miRNAs at a mature miRNA level**. Sequences of PNA inhibitors against individual miRNAs and miR-302a-d, as well as non-targeting controls (NTC) (full length), and a mismatched control (MMC; containing the same nucleotides as miR-302a-d but in a sequence that is not predicted to bind to any segment of the human genome). Note that mmu-miR-294-3p is from the murine miR-290-295 cluster and is the paralog of the human (hsa-) miR-371~373 cluster, and contains the 2-7 nucleotide (2-7nt) seed region AAGUGC (the same as miR-372-3p and miR-373-3p). Otherwise, all mmu- (murine) sequences listed here are identical to their hsa- (human) counterparts. Key: NTC = non-targeting control; MMC = mismatched control.

**Supplementary-Table-S5. Contribution (%) of the miRNA clusters miR-371~373, miR-302-367, and C19MC to 2-7nt AAGUGC abundance in malignant GCT cell lines, as assessed by microarray and qRT-PCR.**

**Supplementary-Table-S6. Cell numbers and percentage of live and dead cells in the malignant GCT cell lines TCam-2 (Sem), 1411H (YST) and 2102Ep (EC) following transfection with miR-302 super family inhibitor (miR-302-SFI), compared with mismatched control (MMC) treated and untreated cells.** Cell numbers as counted using trypan blue dye using the Countess automated cell counter system on d2.

**Supplementary-Table-S7. Identification of de-repressed genes common to both TCam-2 (Sem) and 1411H (YST) cell lines following miR-302-SFI (miR-302 super-family-inhibitor) treatment.** The 188 common seed complementary region (SCR)-containing genes identified by the intersection of the top-ranking 1,600 de-repressed mRNA targets in TCam-2 (881 SCR-containing) and 1411H (775 SCR-containing) treated cells, using the *Sylamer* algorithm on global mRNA expression data performed on day 2 following transfection. Gene names and NCBI gene NM accession numbers are listed. This represents 24.2% overlap between the two SCR-containing gene lists.

**Supplementary-Table-S8. Initial optimisation of cell cycle analysis experiments using the malignant GCT cell line TCam-2 (Sem) with miR-302 super-family-inhibitor (miR-302-SFI) treatment.** Flow cytometry data show the proportion (%) of cells in G0/G1-, G2/M- and S-phase of the cell cycle. Results are also shown for mismatched control (MMC) treated and untreated cells. Experiments were undertaken at d2, d3, and d4 following treatment. The relevant G0/G1- and S-phase comparisons for miR-302-SFI and MMC treated cells are highlighted by a grey background, showing subtle decreases in the proportion of cells in S-phase and increases in the proportion of cells in G0/G1-phase at d2, d3, and d4.

**Supplementary-Table-S9. Definitive cell cycle analysis experiments undertaken on the malignant GCT cell lines TCam-2 (Sem) and GCT44 (YST) cells following short super-family-inhibitor (short-SFI) treatment.** Flow cytometry data show the proportion (%) of cells in G0/G1-, G2/M-, and S-phase of the cell cycle. Results are also shown for mismatched control (MMC) and untreated cells, compared with those obtained with a short (10nt) LNA-based inhibitor (‘short-SFI’). The results indicate that treatment with the short-SFI resulted in a decrease in the proportion of cells in S-phase and an increase in the proportion of cells in G0/G1-phase at d2 and d3.

**Supplementary References**

1. Lin Y, Cradick TJ, Bao G. Designing and testing the activities of TAL effector nucleases. Methods Mol Biol. 2014;1114:203-19.

2. Reljic B, Stroud DA. Screening Strategies for TALEN-Mediated Gene Disruption. Methods Mol Biol. 2016;1419:231-52.

3. Morelli E, Biamonte L, Federico C, Amodio N, Di Martino MT, Gallo Cantafio ME, et al. Therapeutic vulnerability of multiple myeloma to MIR17PTi, a first-in-class inhibitor of pri-miR-17-92. Blood. 2018;132(10):1050-63.

4. Amodio N, Stamato MA, Juli G, Morelli E, Fulciniti M, Manzoni M, et al. Drugging the lncRNA MALAT1 via LNA gapmeR ASO inhibits gene expression of proteasome subunits and triggers anti-multiple myeloma activity. Leukemia. 2018;32(9):1948-57.

5. Rio DC, Ares M, Jr., Hannon GJ, Nilsen TW. Purification of RNA using TRIzol (TRI reagent). Cold Spring Harb Protoc. 2010;2010(6):pdb prot5439.

6. Quijano E, Bahal R, Ricciardi A, Saltzman WM, Glazer PM. Therapeutic Peptide Nucleic Acids: Principles, Limitations, and Opportunities. Yale J Biol Med. 2017;90(4):583-98.

7. Palmer RD, Murray MJ, Saini HK, van Dongen S, Abreu-Goodger C, Muralidhar B, et al. Malignant germ cell tumors display common microRNA profiles resulting in global changes in expression of messenger RNA targets. Cancer Res. 2010;70(7):2911-23.

8. Murray MJ, Saini HK, Siegler CA, Hanning JE, Barker EM, van Dongen S, et al. LIN28 Expression in malignant germ cell tumors downregulates let-7 and increases oncogene levels. Cancer Res. 2013;73(15):4872-84.

9. Liu Q, Wang J, Zhao Y, Li CI, Stengel KR, Acharya P, et al. Identification of active miRNA promoters from nuclear run-on RNA sequencing. Nucleic Acids Res. 2017;45(13):e121.
