# Supplementary figures and images for: Targeting oncogenic microRNAs from the miR-371~373 and miR-302/367 clusters in malignant germ cell tumours causes growth inhibition through cell cycle disruption
Source: Br J Cancer. 2023 Oct 3;129(9):1451–61. doi: 10.1038/s41416-023-02453-1 (PMC10628203; doi:10.1038/s41416-023-02453-1)

Supplementary-Figure-S1

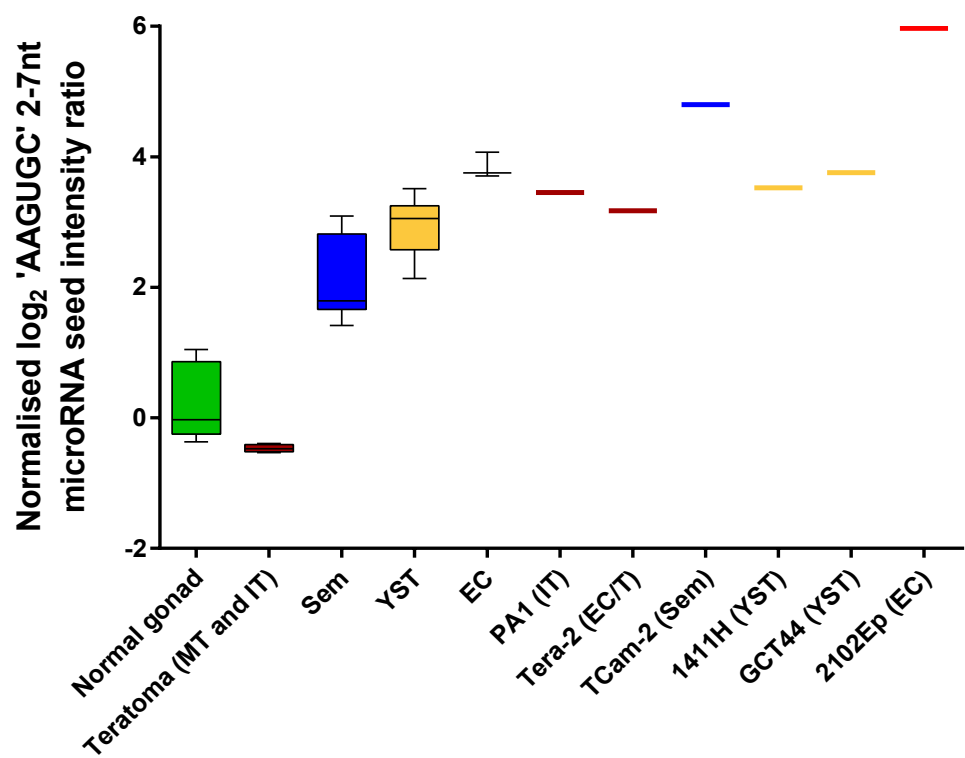

Supplement: Supplementary file 2 — Supplementary Figure S1 [file 41416_2023_2453_MOESM2_ESM.pdf]

### Supplementary-Figure-S.

# A

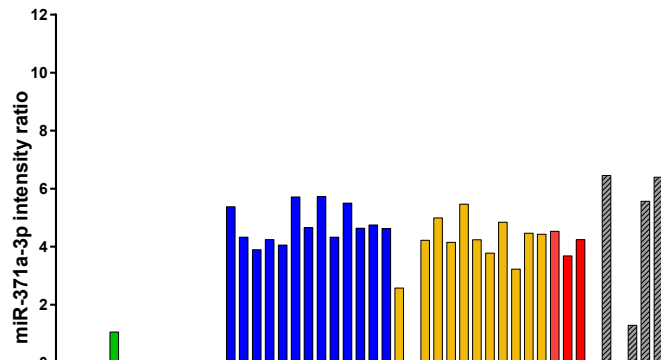

C

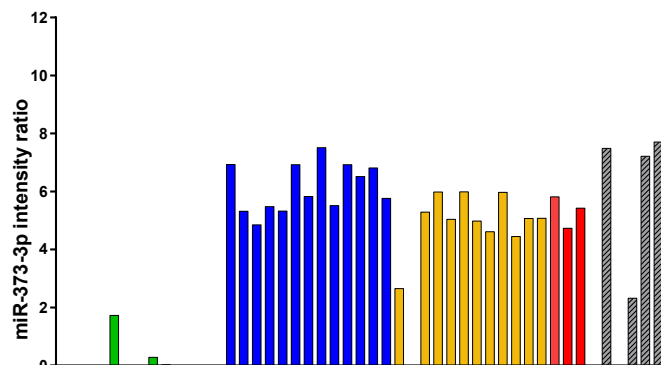

# E

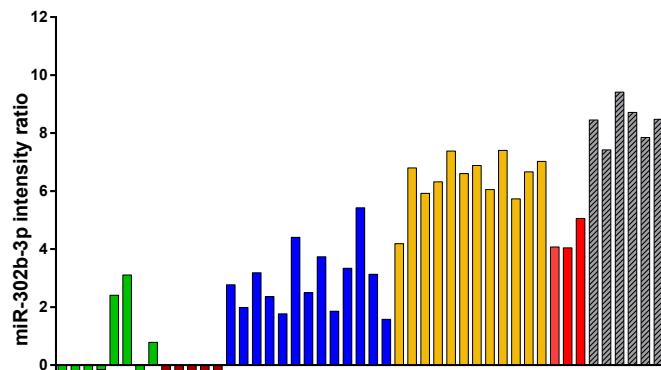

# G

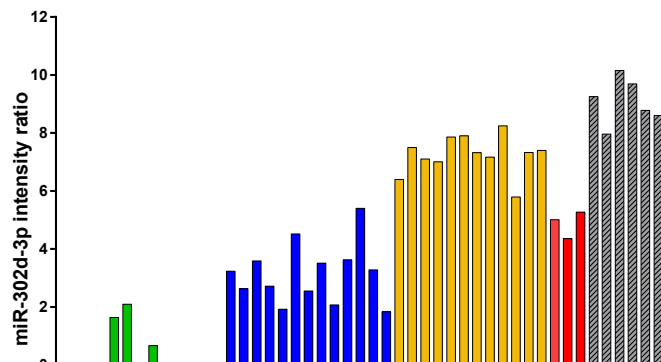

1

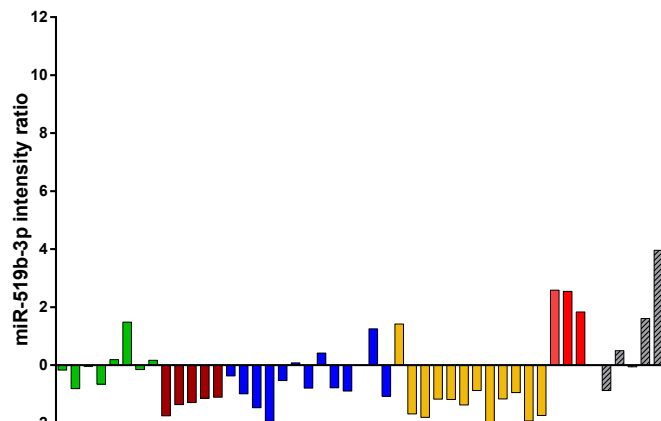

B

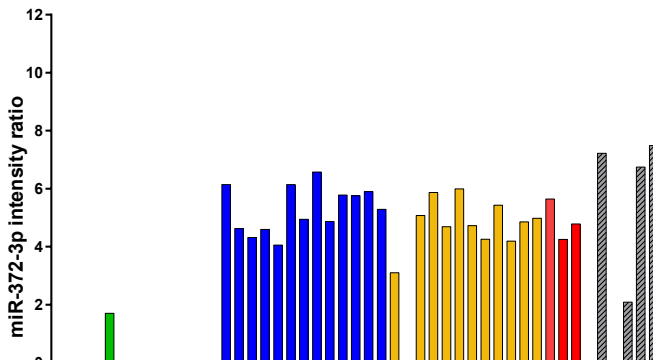

D

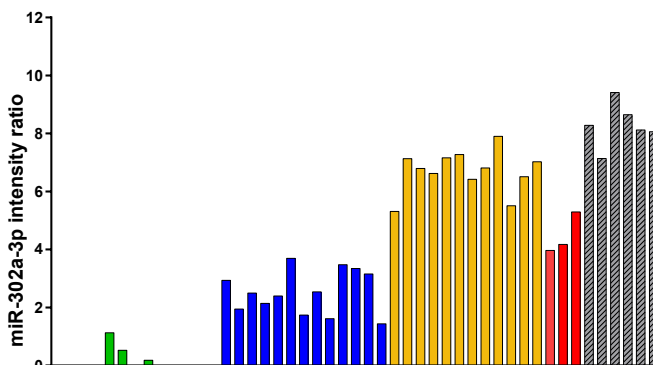

F

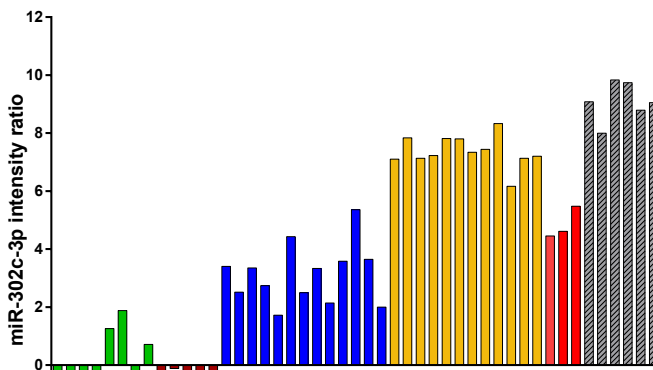

H

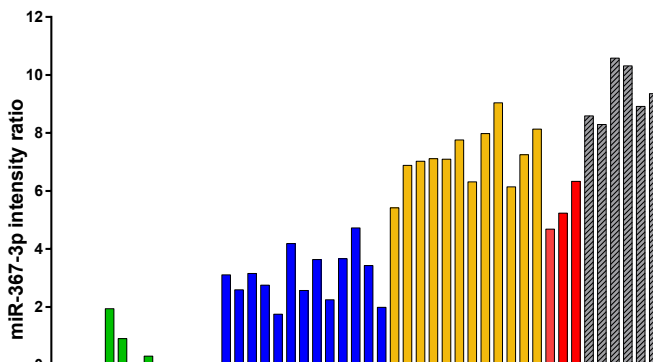

J

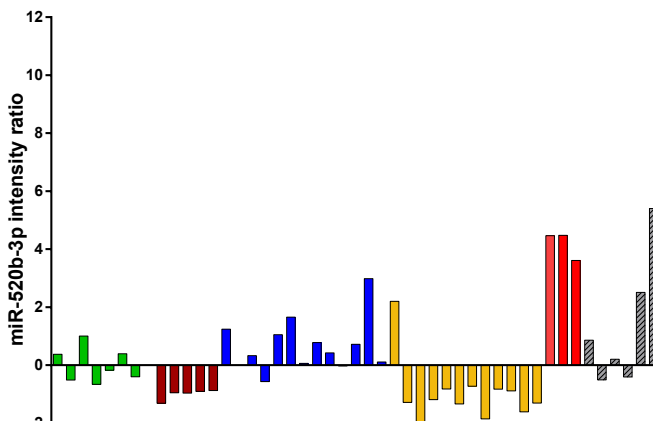

Supplement: Supplementary file 3 — Supplementary Figure S2 [file 41416_2023_2453_MOESM3_ESM.pdf]

# Supplementary-Figure-S3

**A**

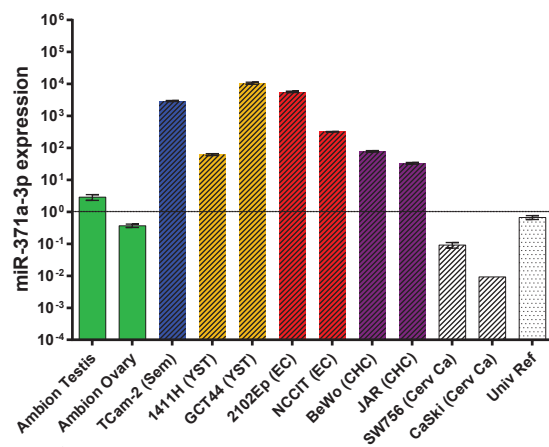

**B**

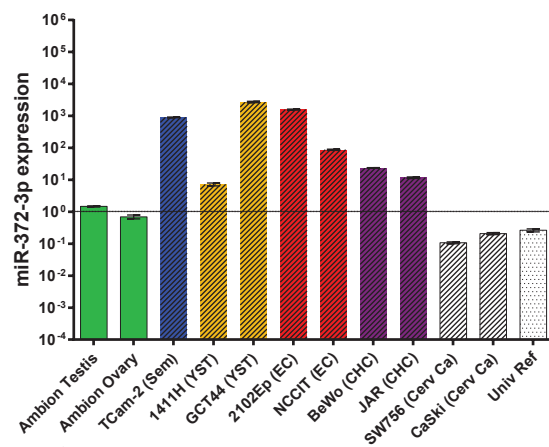

**C**

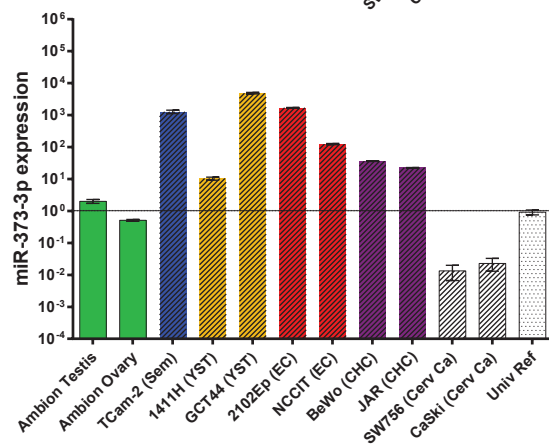

**D**

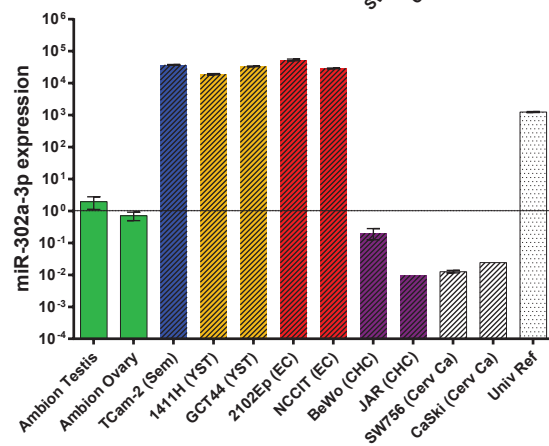

**E**

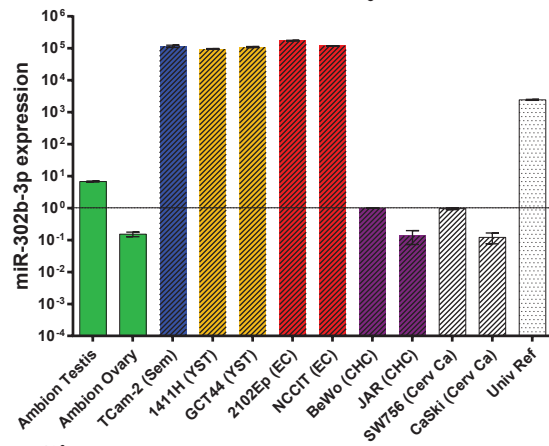

**F**

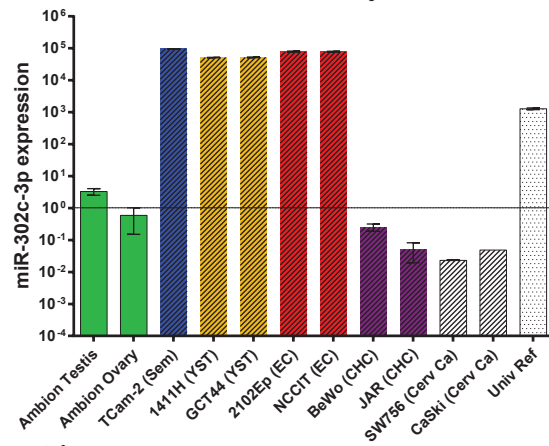

**G**

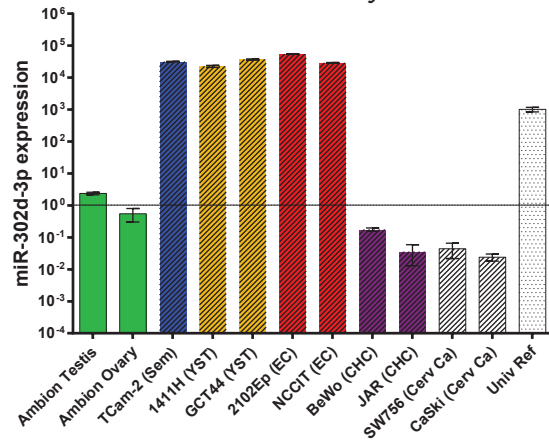

**H**

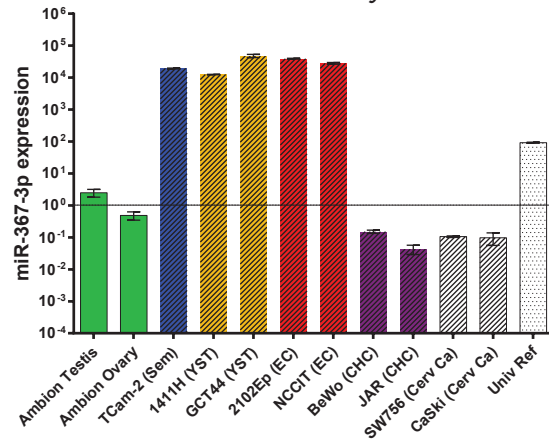

**I**

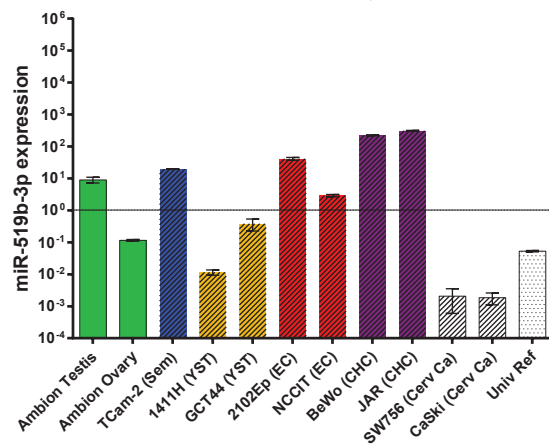

**J**

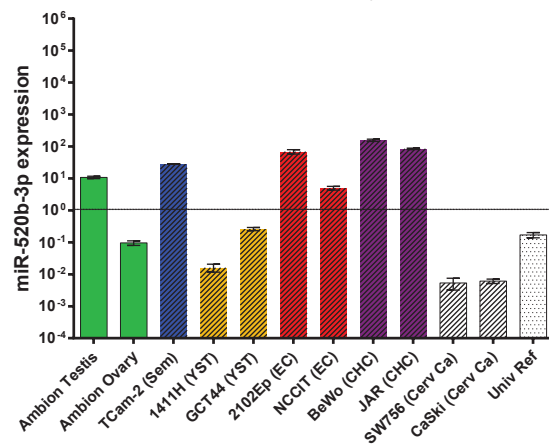

Supplement: Supplementary file 4 — Supplementary Figure S3 [file 41416_2023_2453_MOESM4_ESM.pdf]

Supplementary-Figure-S4

A

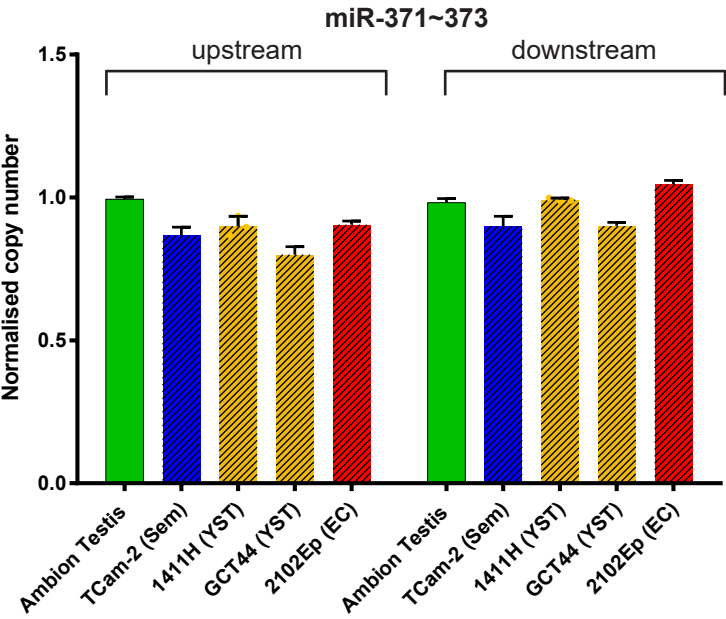

B

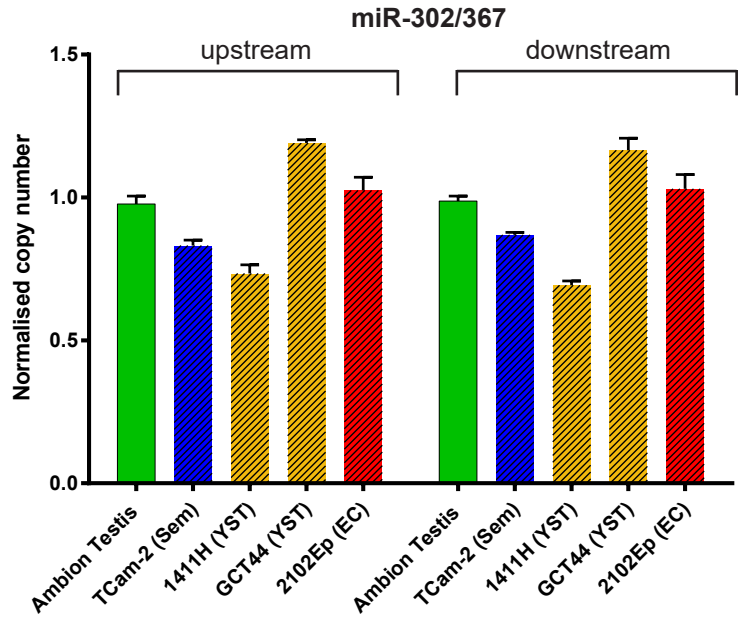

Supplement: Supplementary file 5 — Supplementary Figure S4 [file 41416_2023_2453_MOESM5_ESM.pdf]
